# Comprehensive Pathogen Identification, Antibiotic Resistance, and Virulence Genes Prediction Directly From Simulated Blood Samples and Positive Blood Cultures by Nanopore Metagenomic Sequencing

**DOI:** 10.3389/fgene.2021.620009

**Published:** 2021-03-24

**Authors:** Menglan Zhou, Yarong Wu, Timothy Kudinha, Peiyao Jia, Lei Wang, Yingchun Xu, Qiwen Yang

**Affiliations:** ^1^Department of Clinical Laboratory, Peking Union Medical College Hospital, Peking Union Medical College, Chinese Academy of Medical Sciences, Beijing, China; ^2^Beijing Applied Biological Technologies Co., Ltd., Beijing, China; ^3^School of Biomedical Sciences, Charles Sturt University, Orange, NSW, Australia; ^4^Pathology West, NSW Health Pathology, Orange, NSW, Australia; ^5^Graduate School, Peking Union Medical College, Chinese Academy of Medical Sciences, Beijing, China

**Keywords:** bloodstream infection, MinION nanopore sequencing, identification, resistance, virulence

## Abstract

Bloodstream infection is a major cause of morbidity and mortality worldwide. We explored whether MinION nanopore sequencing could accelerate diagnosis, resistance, and virulence profiling prediction in simulated blood samples and blood cultures. One milliliter of healthy blood samples each from direct spike (sample 1), anaerobic (sample 2), and aerobic (sample 3) blood cultures with initial inoculation of ∼30 CFU/ml of a clinically isolated *Klebsiella pneumoniae* strain was subjected to DNA extraction and nanopore sequencing. Hybrid assembly of Illumina and nanopore reads from pure colonies of the isolate (sample 4) was used as a reference for comparison. Hybrid assembly of the reference genome identified a total of 39 antibiotic resistance genes and 77 virulence genes through alignment with the CARD and VFDB databases. Nanopore correctly detected *K. pneumoniae* in all three blood samples. The fastest identification was achieved within 8 h from specimen to result in sample 1 without blood culture. However, direct sequencing in sample 1 only identified seven resistance genes (20.6%) but 28 genes in samples 2–4 (82.4%) compared to the reference within 2 h of sequencing time. Similarly, 11 (14.3%) and 74 (96.1%) of the virulence genes were detected in samples 1 and 2–4 within 2 h of sequencing time, respectively. Direct nanopore sequencing from positive blood cultures allowed comprehensive pathogen identification, resistance, and virulence genes prediction within 2 h, which shows its promising use in point-of-care clinical settings.

## Introduction

Bloodstream infections (BSIs) are a major cause of morbidity and mortality worldwide. In severe cases, BSI often progresses to sepsis or even septic shock accompanied by multi-organ failure and ends up with death in 30–50% of cases (Engel et al., 2007; Angus and van der Poll, 2013). Accurate pathogen identification and administration of appropriate antibiotic therapy are crucial for the early management of BSI. Each hour of delay in the administration of initial appropriate antimicrobial therapy has been reported to be associated with a 7.6% survival decrease for a septic patient who remains untreated or receives inappropriate antimicrobial therapy within the first 24 h (Zhou et al., 2017). So far, blood culture (BC) remains the gold standard for the diagnosis of BSI but with noticeable limitations. The identification of a pathogen and subsequent antimicrobial susceptibility testing rely largely on the microbial growth, which may take at least 1 day to months (Grumaz et al., 2016; Anson et al., 2018; Ashikawa et al., 2018). Furthermore, this culture-based procedure may yield false-negative results if the patient is given empiric antibiotic therapy before BC is performed (Grumaz et al., 2016). Therefore, early identification of the causative pathogen as well as its antibiotic resistance pattern is the highest priority for improving patient prognosis.

Several culture-independent methods have been introduced to reduce the turnaround time for BSIs. For example, matrix-assisted laser desorption/ionization mass spectrometry-based identification and antimicrobial susceptibility testing methods have been used with varying degrees of success (Morgenthaler and Kostrzewa, 2015; Caspar et al., 2017; Zhou et al., 2017). Microarray and PCR-based molecular techniques have also been introduced for fast identification of pathogens as well as specific drug-resistance markers targeting the causative pathogen from BSI (Ledeboer et al., 2015; Opota et al., 2015; Vardakas et al., 2015). Although these methods can detect pathogens in a relatively short time, none can cover the full antimicrobial resistance pattern of the pathogen involved. Sensitivity and specificity of the methods are largely affected due to the limited panel size, divergence in the primer sequence targeting different species and drug-resistance markers, DNA contamination, and so on, which may contribute to ambiguous results (Chang et al., 2003; Lebovitz and Burbelo, 2013). Thus, there is a constant need to update PCR-based methods so as to include emerging species and antimicrobial resistance genes.

Metagenomic sequencing-based approaches offer a solution to overcome the limitations of both culture- and PCR-based methods, providing fast pathogen species identification, antimicrobial susceptibility prediction, lineage, and other related information based on comprehensive genome data (Loman et al., 2012; Chiu and Miller, 2019). Next-generation sequencing (NGS) platforms, such as Ion Torrent and Illumina, are widely used for metagenomics sequencing, but data analysis cannot begin until the sequencing process is completed and a sufficient read length has been achieved as sequence reads for these platforms are generated in parallel and not in series (Charalampous et al., 2019). The recently developed MinION nanopore sequencer [Oxford Nanopore Technologies (ONT), Oxford, United Kingdom], which is a small, pocket-sized device operated from a laptop connected via USB 3.0, has an advantage of rapid long-reads library preparation and real-time data acquisition over NGS and Sanger sequencing (Ashikawa et al., 2018). To date, limited data are available on the performance of nanopore sequencing to identify BSI pathogens directly from blood samples (Greninger et al., 2015; Anson et al., 2018; Cheng et al., 2018; Sauvage et al., 2018; Taxt et al., 2020). Besides, the platform’s capacity for real-time metagenomic analysis of both resistance and virulence genes directly from samples has not yet been leveraged.

Therefore, in this study, we demonstrate the potential of nanopore sequencing to provide pathogen species identification as well as antimicrobial resistance and virulence genes prediction in a proof-of-concept study using simulated BSI samples.

## Materials and Methods

### Sample Preparation

A clinically isolated *Klebsiella pneumoniae* strain R16 from a liver abscess patient was used in this study. It was a ST11-K47 hypervirulent carbapenem-resistant strain, carrying a rare plasmid (pR16-Hv-CRKp1) harboring *bla*_*KPC–*2_, *bla*_*SHV–*12_, *bla*_*TEM–*1_, *bla*_*CTX–M–*65_, *rmtB*, and a predicted virulence gene R16_5486 simultaneously (Yang et al., 2020). Identification and antimicrobial susceptibility testing were performed using matrix-assisted laser desorption/ionization time-of-flight mass spectrometry (MALDI-TOF MS) and standard broth microdilution (BMD). Simulated BSI samples were prepared by spiking strain R16 into a healthy volunteer blood, each with ∼30 CFU/ml. Ten milliliters of the blood sample was each inoculated into aerobic and anaerobic BC bottles and put into an automated BC machine (BACTEC^TM^ FX400, Becton Dickinson) until flagged positive.

**FIGURE 1:**
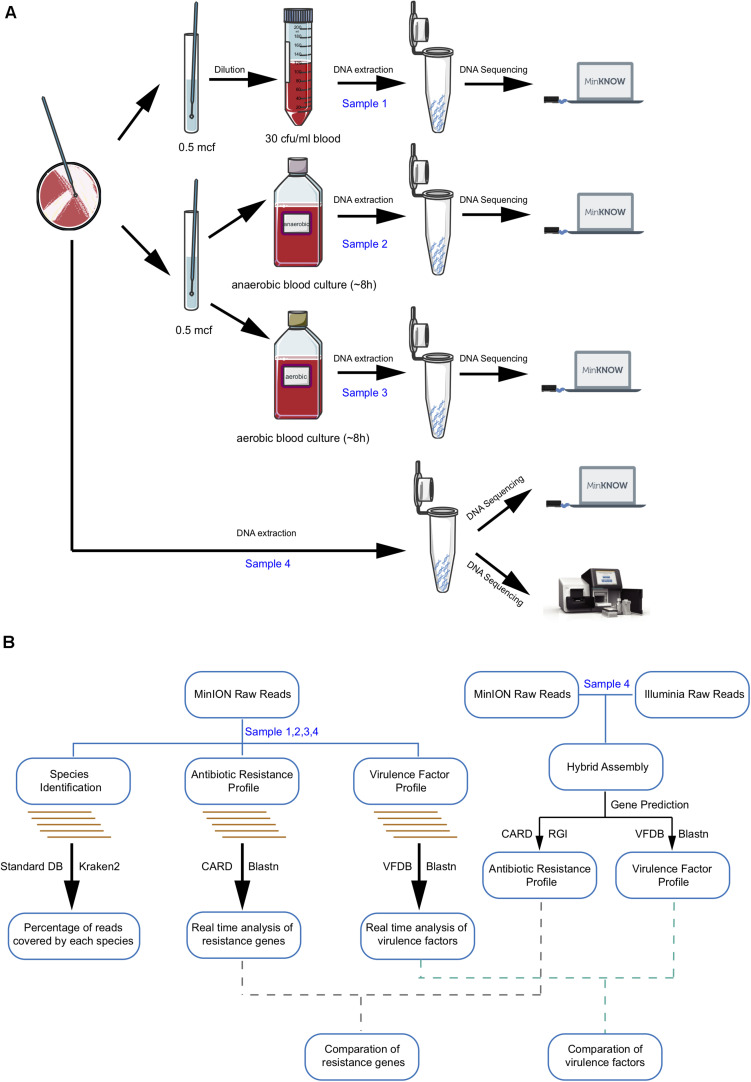
Workflow of laboratory and bioinformatics methods required for metagenomic pathogen detection. **(A)** Sample preparation and sequencing. **(B)** Bioinformatic analysis algorithm. Boxes indicate main analysis steps, and texts on the left side of the arrow show the database used for identification, whereas texts on the right sides indicate the type of tool used.

TIANamp Bacteria DNA Kit (DP302) was used for DNA extraction from 1 ml each of the directly spiked blood sample (sample 1), positive anaerobic BC (sample 2), and positive aerobic BC (sample 3) and from the pure colonies of R16 (sample 4). DNA purification steps were performed with Agencourt AMPure XP Reagent beads (A63880, Beckman). The yield of extracted DNA was quantified and qualified using the NanoDrop 2000 (Thermo Fisher Scientific, Waltham, MA, United States) and Qubit 2.0 Fluorometer (Thermo Fisher Scientific, Waltham, MA, United States). Samples with DNA concentrations higher than 17 ng/μl, OD_260/280_ = 1.8–2.0, and OD_260/230_ ranging between 2.0 and 2.2 were considered acceptable. All four DNA samples were sequenced using an Oxford Nanopore MinION device while sample 4 was also sent for Illumina HiSeq sequencing (Novogene Co., Ltd., Beijing, China) at the same time (Figure 1A).

### Illumina Library Preparation and Sequencing

Illumina Genome Analyzer 2X technology (Illumina, San Diego, CA, United States) was used for genomic DNA extraction and shotgun sequencing. Adapters and low-quality sequences were trimmed and filtered, and shovill v1.0.1^1^ was used for the *de novo* assembly of these reads.

### MinION Library Preparation and Sequencing

For each sample, 700 ng to 1 μg of purified DNA was processed with different barcodes (Native Barcoding Kit, EXP-NBD103) after preparation of the DNA ends and then attached to the sequencing adapters using a Ligation Sequencing kit (SQK-LSK308; ONT) according to the manufacturer’s instructions.

Prior to sequencing, the MinION device was connected to a computer using a USB 3.0 cable and port. A MinION^TM^ R9.4 flow cell was inserted, samples were then loaded, and all sequencing runs were performed for 20 h. Raw electronic signal data were collected and base-called through Albacore v2.2.6^2^. Porechop v0.2.3^3^ was used to remove adaptors and de-multiplex the reads into bins based on which barcode was found. A quality control was run through Pauvre v0.1.86^4^ to generate basic statistics and marginal histogram concerning the fastq files to ensure read quality was within expected boundaries.

### Bioinformatics Analysis

Sequences were further filtered using NanoFilt v2.2.0^5^, removing reads with low average base quality score (<7) or read length <1,000 bp before assembly. Canu v1.6 and nanopolish v0.10.1^6^ were used for nanopore long-read-only assembly and polishing (Koren et al., 2017). Moreover, for sample 4, Unicycler v0.4.5 was also used for hybrid assembly of Illumina reads and nanopore reads, which was used as a reference for long-read-only assembly based and fastq-based comparison among samples 1–4 (Gurevich et al., 2013; Wick et al., 2017). Oxford nanopore raw sequencing data of all four samples and short-read sequence data of sample 4 have been deposited in the National Center for Biotechnology (NCBI) database under BioProject PRJNA663005.

Species identification was performed by Kraken Software v2.0.8-beta (standard database, created on 20190611) (Wood and Salzberg, 2014). Results were presented as Kraken-style reports and Sankey diagram. In case of data loss, all base-calling-passed fastq data (low average base quality score ≥7) were used for species identification analysis.

Antibiotic resistance genes in sample 4 were predicted by aligning hybrid assembly sequence against the CARD database through RGI v4.2.2 (Resistance Gene Identifier) and filtered results with identity less than 75% or length coverage less than 50% (Jia et al., 2017), and prediction results were used as a reference. Real-time analyses were performed by aligning MinION fastq reads to CARD database v3.0.0 through blastn (blast+, v2.2.28), seeking results with identity ≥80%, hit length ≥100 bp, and gene coverage ≥70%. Data from the first 2 h of sequencing were compared every 10 min, and that from 20 h were compared every 30 min with the reference.

Amino acid sequence data file from the hybrid assembly of sample 4 generated from RGI v4.2.2 was blasted against the VFDB protein sequences of the core dataset (v20181113) (Liu et al., 2019). Reliable virulence genes were confirmed if sequence identity >80% and query coverage >80%, which were used as a reference for virulence factors. Meanwhile, MinION reads from samples 1 to 4 were blasted against the VFDB database, and results were screened based on identity ≥80%, hit length ≥100 bp, and gene coverage ≥70%. Data from the first 2 h of sequencing were compared every 10 min, and that from 20 h were compared every 30 min with the reference.

## Results

As shown in Figure 1A, all four samples were sent for nanopore sequencing after DNA extraction. More than 16G fastq files were collected after 20-h sequencing and de-multiplexed based on the barcode. Quality control analysis showed that each sample possessed more than 1 × 10^9^ bp and 450,000 reads with an average length of 3–5 kb, contributing to a mean quality score of 11.5 (Supplementary Table 1 and Supplementary Figure 1). As a real-time sequencing platform, data produced by the MinION system can be base-called and analyzed along with sequencing. Species identification and antibiotic resistance genes and virulence genes analyses were performed based on real-time nanopore sequencing data (Figure 1B).

### Hybrid Assembly of Reference Genome

Hybrid assembly of Illumina reads and Nanopore reads from sample 4 was used as a reference for comparison between the single-assembly results. Assemblies generated a circular chromosome and five plasmid genome sequences all together (Supplementary Table 2). A comparison between Illumina assembly and nanopore assembly revealed that the nanopore long-read-only assembly had a higher genome coverage and much less contigs but higher indels than the Illumina assembly (Supplementary Table 3).

Antibiotic resistance gene analysis based on the hybrid assembly generated 39 resistance genes, including five protein variants/overexpression models and 34 protein homolog models, 32 of which were located on the chromosome while the rest on the plasmid (Supplementary Table 4). All predicted genes were consistent with phenotypic antimicrobial susceptibility profiles (Table 1). Similarly, 77 virulence factors were identified through hybrid assembly alignment against protein sequences in VFDB set A (Supplementary Table 5). All the antibiotic resistance genes and virulence genes predicted from the protein homolog model were considered as reference in subsequent comparison.

**TABLE 1:** Comparison between phenotypic antimicrobial susceptibility profiles and predicted resistance genes.

Antibiotics	Drug class	MIC (mg/L)	Related AMR genes detected by nanopore sequencing	Consistent or not*
Cefotaxime	Third-generation cephalosporin	>32	Beta-lactamase genes (KPC-2, SHV-12, SHV-11, CTX-M-65, CTX-M-45)	Consistent
Ceftriaxone	Third-generation cephalosporin	>32	Beta-lactamase genes (KPC-2, SHV-12, SHV-11, CTX-M-65, CTX-M-45)	Consistent
Ceftazidime	Third-generation cephalosporin	>32	Beta-lactamase genes (KPC-2, SHV-12, SHV-11, CTX-M-65, CTX-M-45)	Consistent
Cefepime	Fourth-generation cephalosporin	>32	Beta-lactamase genes (KPC-2, SHV-12, SHV-11, CTX-M-65, CTX-M-45)	Consistent
Aztreonam	Monobactam	>16	Beta-lactamase genes (KPC-2, SHV-12, SHV-11, CTX-M-65, CTX-M-45, TEM-1)	Consistent
Cefoxitin	Cephamycin	>16	Beta-lactamase genes (KPC-2), efflux genes (H-NS)	Consistent
Ertapenem	Carbapenem	>4	Beta-lactamase genes (KPC-2)	Consistent
Imipenem	Carbapenem	>32	Beta-lactamase genes (KPC-2)	Consistent
Meropenem	Carbapenem	>16	Beta-lactamase genes (KPC-2)	Consistent
Ceftolozane–tazobactam	Beta-lactam/beta-lactamase inhibitor	>32	Beta-lactamase genes (KPC-2)	Consistent
Piperacillin–tazobactam	Beta-lactam/beta-lactamase inhibitor	>64	Beta-lactamase genes (KPC-2)	Consistent
Ceftazidime–avibactam	Beta-lactam/beta-lactamase inhibitor	1	–	Consistent
Imipenem–relebactam	Carbapenem/carbapenemase inhibitor	0.5	–	Consistent
Ciprofloxacin	Fluoroquinolone	>2	Efflux (*oqxAB*, *patA*, *emrBR*, CRP, *acrAB*, *mdtABC*, H-NS), target alteration (*gyrA*, *parC*)	Consistent
Polymyxin	Lipopeptide	≤1	–	Consistent
Amikacin	Aminoglycoside	>32	*aadA*, *AAC(6′)-IIb*, *rmtB*, *baeR*, *mdtABC*	Consistent
Tetracycline	Tetracycline	>32	Efflux (*mdfA*, *acrAB*, *tet*, *oqxAB*, H-NS)	Consistent

*Represents all predicted genes were consistent with phenotypic antimicrobial susceptibility profiles.

### Species Identification

Analysis using the Kraken database was performed on three samples for species identification. In all cases, nanopore sequencing correctly detected *K. pneumoniae* but with varied sensitivity. For sample 1, in which targeted species enrichment was not performed, *K. pneumoniae*-related sequences only accounted for 0.05% of the total reads. The majority of the reads were from *Homo sapiens* (98.49%) (Supplementary Table 6). Among all the reads generated from sample 1, the longest one was 25,644 bp, which yielded a 99% coverage and 87% consistency with *K. pneumoniae* strain 301 plasmid pKP301b genome (GenBank No. KY354306.1), followed by an 18,609 bp read, which had a 100% coverage and 92% consistency with *K. pneumoniae* chromosome genome of many strains (GenBank Nos. CP041373.1, CP044258.1, CP028583.2, etc.).

However, for samples 2 and 3, which underwent anaerobic and aerobic BC, direct nanopore sequencing detected 65.66% and 62.01% of *K. pneumoniae*-related sequences in the total reads. Besides, the *H. sapiens* reads were much less (sample 2: 7.34%; sample 3: 2.09%) than those in sample 1 (Supplementary Table 6 and Supplementary Figure 2).

**FIGURE 2:**
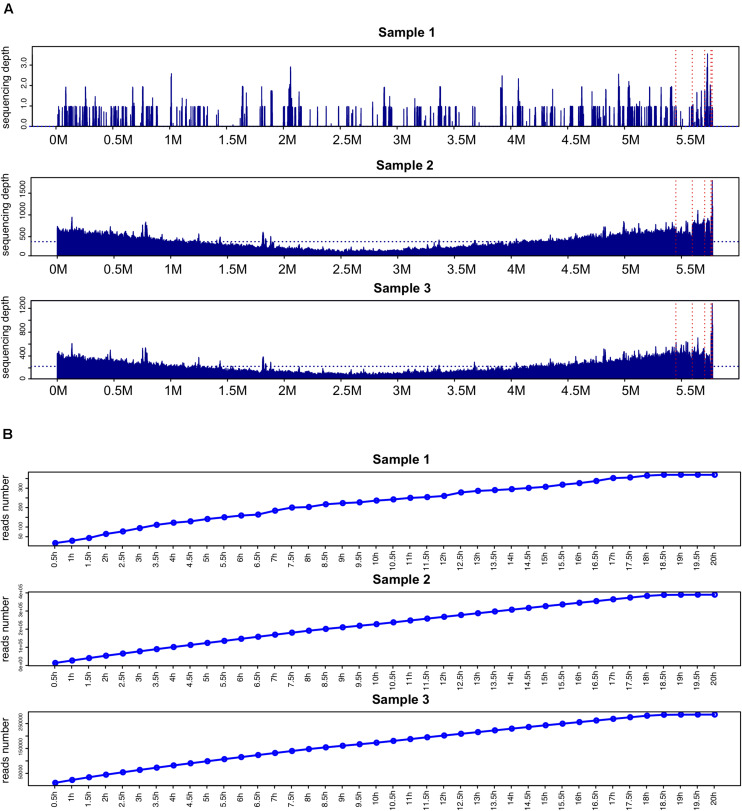
Comparison of genome coverage and reads number of *K. pneumoniae*. **(A)** Comparison of sequencing depth and coverage in samples 1–3. The horizontal blue dashed line indicates the average sequencing depth mapped to the *K. pneumoniae* hybrid assembly genome. The chromosome genome and five plasmid sequences are separated by vertical red dashed lines. **(B)** *K. pneumoniae*-related reads detected in samples 1–3 at different time points.

Comparison of sequencing depth and coverage in the three samples revealed that the genome coverage of *K. pneumoniae* was far less than 17.4% in the unenriched sample 1 while the BC-enriched samples 2 and 3 had a 100% coverage (Figure 2A).

Time-point reads analysis was performed in the three samples (Figure 2B). After sequencing for 2 h, 64, 53,278, and 44,521 *K. pneumoniae*-related reads were detected in samples 1, 2, and 3, respectively. Two reliable *K. pneumoniae*-related reads were detected after sequencing started for 3.5 and 10.4 min in sample 1 (Supplementary Table 7).

### Identification of Antibiotic Resistance Genes

**FIGURE 3:**
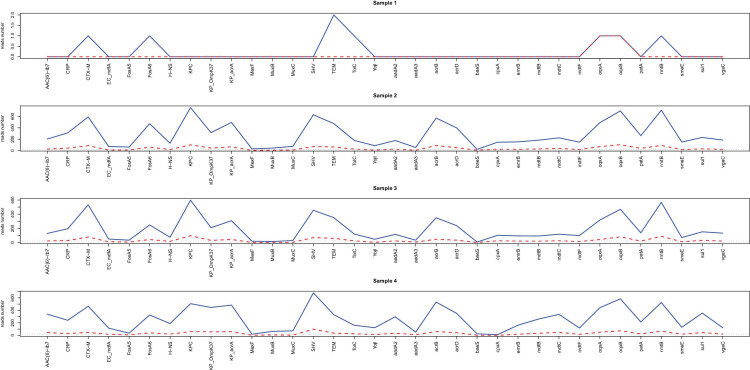
Line chart for the reads number of resistance genes readily identified from nanopore outputs. The acquired resistance genes in samples 1–4 through alignment with the CARD database are shown in the *x*-axis. The reads numbers in different resistance genes for 2 h (red) and 20 h (blue) are provided by the *y* values.

Acquired resistance genes were readily identified from nanopore outputs in samples 1–4 through alignment with the CARD database. Figure 3 shows the number of resistance genes-related reads after sequencing for 2 h (red) and 20 h (blue). As illustrated, more than 20 resistance genes-related reads can be generated from all the samples within 2 h except for sample 1.

Heatmaps of resistance genes-related reads were constructed based on the number of reads calculated every 30 min (Supplementary Figure 3). Compared to those resistance genes identified from the hybrid assembly of sample 4, direct nanopore sequencing correctly identified 7 out of 34 resistance genes for sample 1 (20.6%) and 28 resistance genes in samples 2–4 (82.4%) belonging to the same gene family after sequencing for 20 h.

For samples 2–4, the same number of resistance genes can be identified after sequencing for 2 h with more than 20 supportive reads while for sample 1, only two resistance genes were identified after 2 h sequencing with only one or two supportive reads (Supplementary Figure 4 and Supplementary Table 8). The high consistency among samples 2, 3 and 4 suggested that direct nanopore sequencing of positive BC can be used for resistance genes prediction.

### Identification of Virulence Genes

**FIGURE 4:**
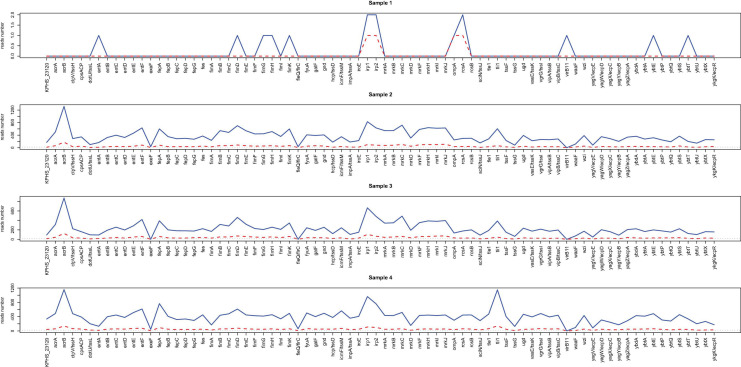
Line chart for the reads number of virulence factors readily identified from nanopore outputs. The acquired virulence genes in samples 1–4 through alignment with the VFDB database are shown in the *x*-axis. The reads numbers in different virulence genes for 2 h (red) and 20 h (blue) are provided by the *y* values.

The virulence gene profiles of the samples were characterized with the nanopore sequencing data in samples 1–4 through alignment with the VFDB database. Figure 4 shows the number of virulence genes-related reads detected after sequencing for 2 h (red) and 20 h (blue). Similar to what was found for resistance genes, more than 20 virulence genes-related reads can be detected from all the samples within 2 h of incubation except for sample 1.

Supplementary Figure 5 illustrates the heatmaps based on the number of virulence genes-related reads detected every 30 min. After sequencing for 20 h, only 12 virulence genes were detected in sample 1, and 11 (14.3%) of them were consistent with the reference. However, 75–77 virulence genes were predicted in samples 2–4 through alignment with the database, and 74 (96.1%) of them were consistent with the reference (Supplementary Table 8).

Besides, for samples 2–4, virulence genes can be detected within 2 h, each of which had more than 20 supportive reads and showing a high consistency with the reference, suggesting that direct nanopore sequencing of positive BC can also be used for virulence genes prediction.

**FIGURE 5:**
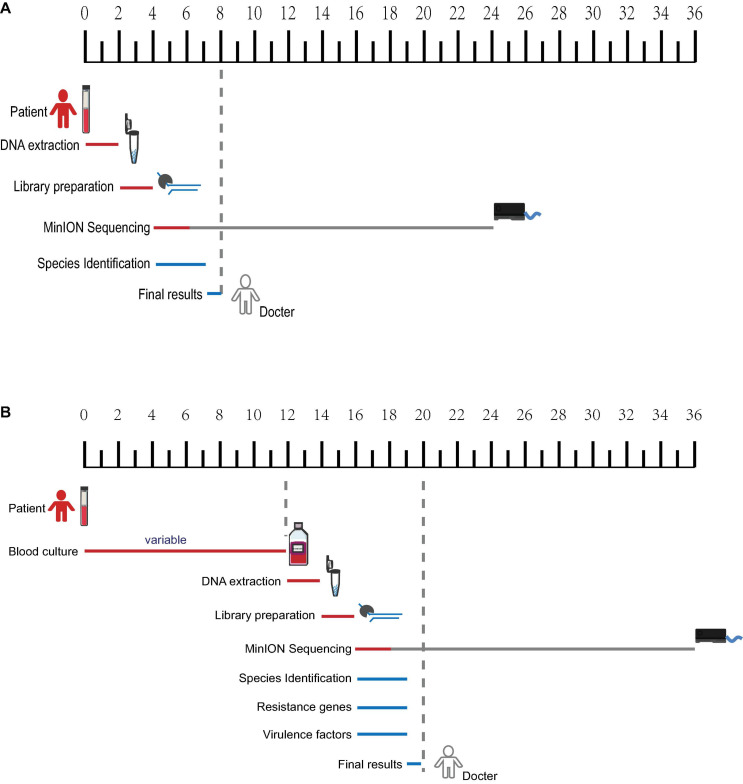
Timeframe diagram for real-time diagnostics of blood samples using nanopore metagenomic sequencing. **(A)** Species identification can be available within 8 h from untreated infectious blood. **(B)** A comprehensive report including species identification, resistance, and virulence genes characterization from blood culture samples can be available within 20 h.

Overall, only dozens of *K. pneumoniae*-related reads could be detected through direct nanopore sequencing with untreated infectious blood, which is useful in species identification but far from being enough for resistance and virulence genes characterization. The identification report can be available within 8 h from the initial blood sampling (Figure 5A). The BC samples can generate 40–50 thousand of related reads after sequencing for 2 h and can be used for species identification, resistance, and virulence genes prediction. The final comprehensive report can be available within 20 h (Figure 5B).

## Discussion

For years, culture-based pathogen identification and susceptibility testing-based tailored antibiotic treatment have been the golden rule for appropriate clinical management of BSI (Grumaz et al., 2016; Anson et al., 2018). However, this culture-dependent method relies largely on the pathogen growth on selective media, which may fail due to the fastidious nature or very low rates of viable microorganisms in the bloodstream or when the patient has been prescribed with antibiotics (Grumaz et al., 2016). All the factors could contribute to a low sensitivity of positive BCs in patients suffering from severe sepsis or septic shock despite the obviously manifested infection symptoms (Schmitz et al., 2013). The recently developed MinION^TM^ device is a promising alternative for diagnosis of BSI by applying long-read single-molecule sequencing directly to clinical samples (Sauvage et al., 2018). So far, several studies have reported using nanopore sequencing metagenomic analysis for species identification from blood (Greninger et al., 2015; Quick et al., 2016; Anson et al., 2018; Ashikawa et al., 2018; Sauvage et al., 2018; Sakai et al., 2019), urine (Schmidt et al., 2017), feces (Leggett et al., 2017), and valves (Cheng et al., 2018). In this study, we sought to demonstrate the potential of the nanopore instrument for metagenomic pathogen identification, as well as antimicrobial drug resistance and virulence genes prediction directly from simulated BSI samples.

It is known that NGS technology such as Illumina is featured in parallel short-reads library and has a very high base-calling accuracy, while third-generation nanopore highlights long-reads sequencing but inevitably renders higher errors in assembly (Cao et al., 2016). Short-read output with high accuracy may be complemented by that produced by much longer reads. So we combined the two to generate a hybrid assembly of Illumina and nanopore for sample 4 (pure colonies of R16) as the reference genome. Three simulated BSI blood samples (two from BC) were subject to nanopore sequencing, and the results were compared with the reference.

Due to the capacity of real-time sequence analysis, we assessed the minimum sequencing time to be able to accurately identify the pathogen and predict resistance genes and virulence genes using nanopore sequencing. *K. pneumoniae* was identified in all three samples, but supportive reads were much less in sample 1 than in samples 2 and 3. Noticeably, *H. sapiens* accounted for the most reads percentage in sample 1, while in samples 2 and 3, the number of *H. sapiens*-related reads was significantly reduced. Nevertheless, two reliable *K. pneumoniae*-related reads were detected after sequencing started for 3.5 and 10.4 min in sample 1. This suggests that fast pathogen identification can be achieved through direct blood nanopore sequencing.

For antibiotic resistance and virulence genes analysis, the extensiveness of pathogen genome coverage was very critical. The advantage of real-time long-reads sequencing of the nanopore technology not only includes facilitating the process of high-quality genome assembly but can also give context to the position of resistance genes and resolve repetitive regions accurately, compared to NGS-based short-reads data (Ashton et al., 2015; Lu et al., 2016; Schmidt et al., 2017). As is shown in this study, the hybrid assembly alignment identified a total of 39 resistance genes, 32 of which were located on the chromosome while the remaining seven were on the plasmid in the reference genome. Noticeably, five of them contributed to resistance through variants/overexpression models and were not considered for subsequent comparison.

Most of the resistance genes (*n* = 28) were identified in samples 2–4, while a very limited number (*n* = 2) was identified in sample 1 after sequencing for 2 h compared to the reference. Extending sequencing time from 2 to 20 h did not result in the identification of more resistance genes in samples 2–4 but identified five more gene families in sample 1, suggesting that 2 h nanopore sequencing of positive BC was sufficient to predict most of the resistance genes. However, all the antibiotic resistance prediction work in this study was based on the CARD antibiotic resistance database, which is limited to genes that confer resistance to antibiotics *per se* (Grumaz et al., 2016). Other diverse resistance mechanisms such as chromosomal point mutations, the resulting gene expression changes due to frame shifts or early truncations and posttranslational modifications, are difficult to distinguish by nanopore sequencing (Ashikawa et al., 2018; Tamma et al., 2019). This makes comprehensive phenotype–genotypic comparisons difficult due to the limited sensitivity of resistance prediction, highlighting the importance of active curation of the resistance gene database used for genotypic prediction (Ellington et al., 2017). In the long run, even achieving completely accurate identification of all resistance genes is only a first step in fully predicting the resistance profile, and complementation with gene expression data such as RNA sequencing may help in elucidating additional resistance mechanisms (Grumaz et al., 2016; Ellington et al., 2017).

Apart from resistance, virulence of a BSI pathogen is also an important factor clinicians may be concerned about when considering appropriate treatment. Early identification of the virulence profiles is crucial for characterizing and tracking pathogenic bacteria as in certain cases, the presence of a virulence gene/plasmid would directly contribute to an infection outbreak (Brynildsrud et al., 2018). For example, the 2011 outbreak of *Escherichia coli* O104:H4 in Germany was due to the presence of the novel *stx* gene (Frank et al., 2011); an unusual outbreak of tuberculosis was caused by *Mycobacterium bovis*, in which genome analysis revealed the presence of an insertion sequence resulting in the upregulation of a number of virulence genes (Gonzalo-Asensio et al., 2014); and a fatal outbreak of ST11 carbapenem-resistant hypervirulent *K. pneumoniae* was found to be associated with the acquisition of a roughly 170 kb pLVPK-like virulence plasmid (Gu et al., 2018). So far, virulence prediction using WGS data was culture based (Brynildsrud et al., 2018; Gonzalez-Escalona et al., 2019). To the best of our knowledge, this is the first study to use direct nanopore sequencing data from samples for virulence prediction.

The clinical strain used in this study for simulated BSI analysis was a hypervirulent *K. pneumoniae*, which was confirmed in our previous experiment by a bacteriological test, neutrophil killing assay, and *Galleria mellonella* infection model (Yang et al., 2020). The reference hybrid assembly results predicted a total of 77 virulence genes through alignment with the VFDB database. The direct nanopore sequencing in simulated samples for virulence genes prediction revealed a similar finding as in resistance. The number of virulence genes identified was dependent on the reads generated. Sample 1 showed the least yield with only 11 virulence genes (14.3%) identified, and extension of sequencing hours to 20 h did not help much, while in samples 2–4, most (74/77, 96.1%) of the virulence genes were able to be identified within 2 h. Based on these congruent findings, it was reasonable for us to believe that nanopore sequencing of positive BC can reliably identify the majority of the pathogen-related virulence genes. Nevertheless, novel virulence genes or even a gene loss-resulting virulence profile cannot be achieved through alignment with the VFDB database due to the limitation of the database itself (Pearson et al., 2016; Willemse et al., 2016). Understanding the pathogenic potential of a pathogen is a complex task with much to be explored in the future.

The timeliness of appropriate antimicrobial therapy is crucial to the reduction of BSI-related morbidity and mortality. The advantages of rapid turnaround time from sample to results and high accessibility to the small portable device makes it possible to use nanopore sequencing technology in clinical point-of-care applications, potentially improving health care strategies and antibiotic stewardship in severe septic patients in the future (Leggett et al., 2017). However, currently, the nanopore sequencing cost is still prohibitively high compared to conventional methods, which remains a major obstacle to the wide use of this technology outside well-resourced research-focused laboratories (Tamma et al., 2019). With the continuous evolvement in the science of WGS, it is promising to see the cost of nanopore runs further reduced by developing cheaper alternative reagents, new public bioinformatic tools, databases for analyzing, and so on (Tamma et al., 2019).

This study has several limitations. First, despite 0.05% *K. pneumoniae*-related reads detected from sample 1, this could be partially affected by the high pathogen concentration in samples 2 and 3 due to the lack of a negative control in the laboratory setting. Second, we provided here only the simulated sample data; clinical positive BC samples in the real word would be tested in our future plan.

In conclusion, we demonstrated that DNA of sufficient quantity and quality extracted from positive BC enabled MinION-based WGS for pathogen identification, resistance, and virulence genes detection within 2 h. A higher sequencing output was shown to correlate with increased sensitivity in pathogen identification and increased confidence in resistance and virulence prediction given that direct sequencing of blood sample without enrichment yielded a much poorer result. Nevertheless, our work represents a proof-of-concept study, and validation needs to be done in more clinical BSI samples in the future.

## Data Availability Statement

The genome sequences reported in this paper have been deposited in the National Center for Biotechnology (NCBI) database under BioProject PRJNA663005.

## Ethics Statement

The studies involving human participants were reviewed and approved by the Institutional Review Board of Peking Union Medical College Hospital (No. S-263). Written informed consent for participation was not required for this study in accordance with the national legislation and the institutional requirements.

## Author Contributions

QY and YX conceived and designed the work. YW, LW, and PJ performed the experiment. MZ analyzed the data and wrote the manuscript. TK reviewed the manuscript and polished the language. All authors read and approved the final manuscript.

## Conflict of Interest

YW and LW were employed by the Beijing Applied Biological Technologies Co., Ltd., Beijing, China. The remaining authors declare that the research was conducted in the absence of any commercial or financial relationships that could be construed as a potential conflict of interest.

## Footnotes

1 https://github.com/tseemann/shovill

2 https://community.nanoporetech.com/downloads

3 https://github.com/rrwick/Porechop

4 https://github.com/conchoecia/pauvre

5 https://pypi.org/project/NanoFilt/

6 https://github.com/jts/nanopolish

## Supplementary Material

Supplementary Figure 1 Scatter plots with marginal histograms for quality control analysis. The *x*-axis represents the phred quality distribution, and the *y*-axis represents the reads length distribution. All four samples possessed more than 1 × 10^9^ bp and 450,000 reads with an average length of 3–5 kb, contributing to a mean quality score of 11.5.

Supplementary Figure 2 Sankey plots for kraken reports. **(a)** Sample 1 detected 0.48% of *K. pneumoniae* related sequences in total reads; **(b)** Sample 2 detected 67.62% of *K. pneumoniae* related sequences in total reads (Sample 3 detected 64.42% of *K. pneumoniae* related sequences in the total reads, similar to Sample 2, data not shown).

Supplementary Figure 3 Heatmaps of resistance genes within 20 h of sequencing. The heatmap displays the total number of reads readily identified from Nanopore outputs in four samples aligned with the CARD database every 30 min since the sequencing started. The bar on the left represents common resistance mechanisms, and the text on the right shows specific gene names with different colours representing different gene families. The dots next to heatmap display the comparison between the resistance genes identified from direct Nanopore sequencing and those identified from hybrid assembly. Red colour suggests the same genes are identified, blue colour means the same gene families are identified and white colour means these genes are not found in Nanopore outputs.

Supplementary Figure 4 Heatmaps of resistance genes within 2 h of sequencing. The heatmap displays the total number of reads readily identified from Nanopore outputs in four samples aligned with the CARD database every 0.1 h since sequencing started. The bar on the left represents common resistance mechanisms, and the text on the right shows specific gene names with different colours representing different gene families. The dots next to heatmap display the comparison between the resistance genes identified from direct Nanopore sequencing and those identified from hybrid assembly. Red colour suggests the same genes are identified, blue colour means the same gene families are identified and white colour means these genes are not found in Nanopore outputs.

Supplementary Figure 5 Heatmaps of virulence factors within 20 h of sequencing. The heatmap displays the total number of reads readily identified from Nanopore outputs in four samples aligned with the VFDB database every 30 min since sequencing started. The bar on the left represents common classifications of virulence factors, and the text on the right shows specific virulence gene names. The dots next to heatmap display the comparison between the virulence genes identified from direct Nanopore sequencing and those identified from hybrid assembly. Red colour suggests the same genes are identified, and white colour means these genes are not found in Nanopore outputs.

Supplementary Figure 6 Heatmaps of virulence factors within 2 h of sequencing. The heatmap displays the total number of reads readily identified from Nanopore outputs in four samples aligned with the VFDB database every 0.1 h since sequencing started. The bar on the left represents common classifications of virulence factors, and the text on the right shows specific virulence gene names. The dots next to heatmap display the comparison between the virulence genes identified from direct Nanopore sequencing and those identified from hybrid assembly. Red colour suggests the same genes are identified, and white colour means these genes are not found in Nanopore outputs.
