# Supplementary figures and images for: Comprehensive Pathogen Identification, Antibiotic Resistance, and Virulence Genes Prediction Directly From Simulated Blood Samples and Positive Blood Cultures by Nanopore Metagenomic Sequencing
Source: Front Genet. 2021 Mar 24;12:620009. doi: 10.3389/fgene.2021.620009 (PMC8024499; doi:10.3389/fgene.2021.620009)

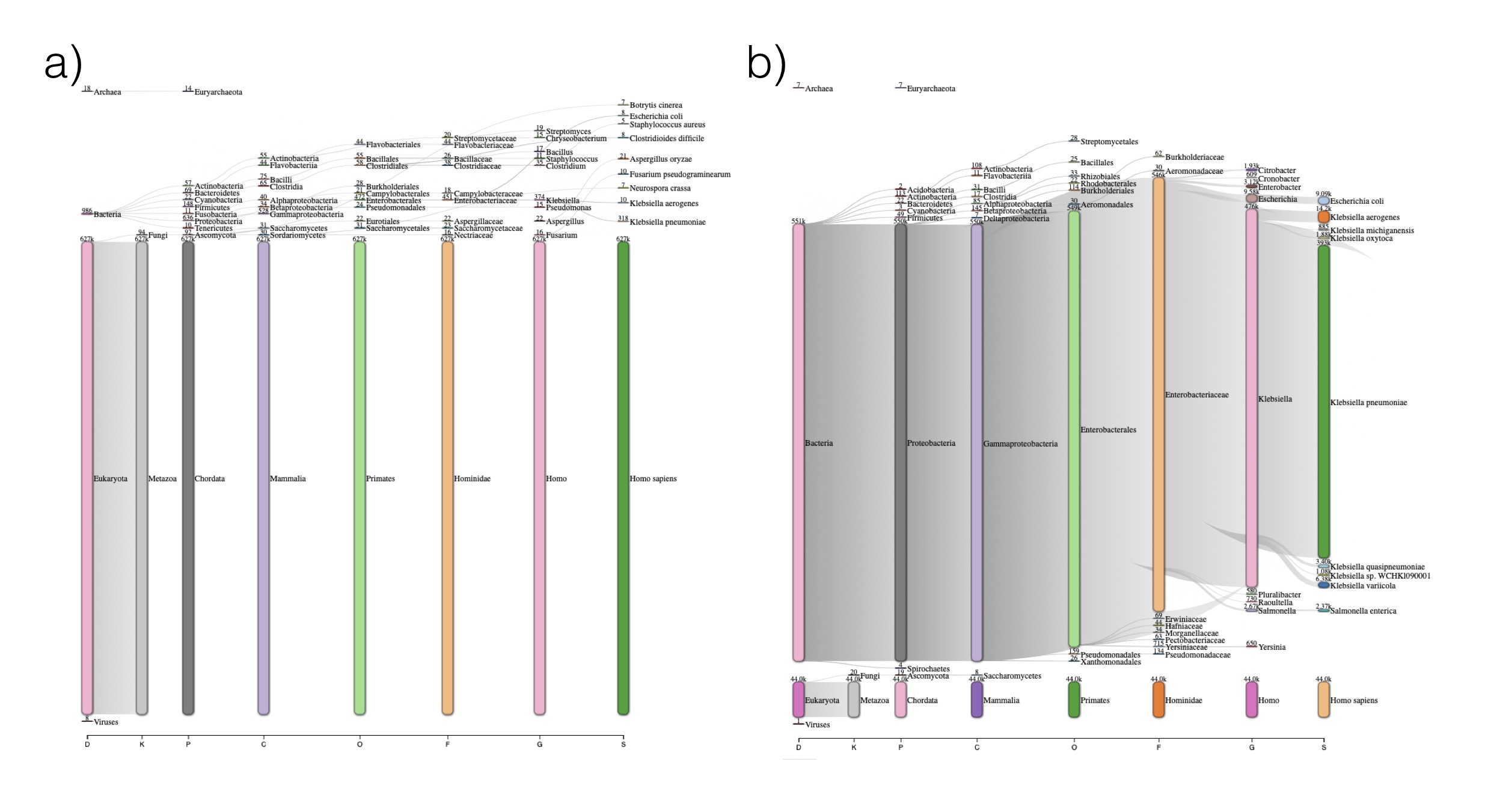

Supplement: Supplementary Figure 2 — Sankey plots for kraken reports. (a) Sample 1 detected 0.48% of K. pneumoniae related sequences in total reads; (b) Sample 2 detected 67.62% of K. pneumoniae related sequences in total reads (Sample 3 detected 64.42% of K. pneumoniae related sequences in the total reads, similar to Sample 2, data not shown). [file Image_2.JPEG]
