# Supplementary material for: Comprehensive Pathogen Identification, Antibiotic Resistance, and Virulence Genes Prediction Directly From Simulated Blood Samples and Positive Blood Cultures by Nanopore Metagenomic Sequencing
Source: Front Genet. 2021 Mar 24;12:620009. doi: 10.3389/fgene.2021.620009 (PMC8024499; doi:10.3389/fgene.2021.620009)
Supplement: Supplementary file 7 [file Table_1.DOCX]

**Supplementary Tables**

Supplementary Table S1. Summary of Nanopore sequencing results for all four samples.

| Sample ID | Sample type | Input  (ng) | Base pairs (bp) | No. of reads | Mean length (bp) | Max length (bp) |
| --- | --- | --- | --- | --- | --- | --- |
| Sample 1 | Simulated blood infection | 795.8 | 1,836,446,176 | 636,356 | 2885.88 | 45,026 |
| Sample 2 | Anaerobic blood culture | 1324.8 | 2,412,679,513 | 599,069 | 4027.38 | 81,288 |
| Sample 3 | Aerobic blood culture | 1748 | 1,386,018,901 | 458,088 | 3025.66 | 59,725 |
| Sample 4 | Isolate | 1770.8 | 2,196,832,617 | 423,473 | 5187.66 | 86,557 |

No. number

Supplementary Table S2. Hybrid assembly results of Illumina reads and Nanopore reads for sample 4.

| Seq* | Type | Length (bp) | Circulator |
| --- | --- | --- | --- |
| 1 | Chromosome | 5450686 | Yes |
| 2 | Plasmid | 144820 | Yes |
| 3 | Plasmid | 109507 | Yes |
| 4 | Plasmid | 55186 | Yes |
| 5 | Plasmid | 10060 | Yes |
| 6 | Plasmid | 5596 | Yes |

*Seq: The number represents chromosome or plasmid genome.

Supplementary Table S3. QUAST comparison of single assembly of Illumina only reads and Nanopore only reads to hybrid assembly of both Illumina and Nanopore reads of sample 4.

| Genome statistics | Illumina only  (%) | Nanopore only  (%) | Hybrid assembly  (%) |
| --- | --- | --- | --- |
| Genome fraction (%) | 98.299 | 99.964 | 100 |
| Duplication ratio | 1.001 | 1.015 | 1 |
| Largest alignment | 387403 | 5046802 | 5450686 |
| Total aligned length | 5685023 | 5859193 | 5775855 |
| NGA50 | 176134 | 5046802 | 5450686 |
| LGA50 | 12 | 1 | 1 |
| Misassemblies | 0 | 6 | 0 |
| Misassembled contigs length | 0 | 5804969 | 0 |
| Mismatches per 100 kbp | 0.63 | 130.94 | 0 |
| Indels per 100 kbp | 0.48 | 268.7 | 0 |
| N's per 100 kbp | 0 | 0 | 0 |
| Statistics without reference | |  |  |
| Contigs | 171 | 6 | 6 |
| Largest contig | 387403 | 5480383 | 5450686 |
| Total length | 5688162 | 5860171 | 5775855 |
| Total length (>= 1000 bp) | 5653499 | 5860171 | 5775855 |
| Total length (>= 10000 bp) | 5530427 | 5860171 | 5770259 |
| Total length (>= 50000 bp) | 5043866 | 5838196 | 5760199 |

Supplementary Table S4. Thirty-nine antimicrobial resistance genes identified based on hybrid assembly of sample 4.

| Seq^*^ | Besthit ARO | AMR gene family | Identity  (%) | Percent  (%) | Cut off | Model type |
| --- | --- | --- | --- | --- | --- | --- |
| 2 | CTX-M-65 | CTX-M beta-lactamase | 100 | 100 | Perfect | homolog |
| 1 | SHV-11 | SHV beta-lactamase | 100 | 100 | Perfect | homolog |
| 1 | *oqxA* | resistance-nodulation-cell division (RND) antibiotic efflux pump | 100 | 100 | Perfect | homolog |
| 1 | *sul1* | sulfonamide resistant *sul* | 100 | 68.46 | Strict | homolog |
| 2 | SHV-12 | SHV beta-lactamase | 100 | 102.51 | Perfect | homolog |
| 2 | TEM-1 | TEM beta-lactamase | 100 | 100 | Perfect | homolog |
| 2 | KPC-2 | KPC beta-lactamase | 100 | 100 | Perfect | homolog |
| 2 | *rmtB* | 16S rRNA methyltransferase (G1405) | 100 | 100 | Perfect | homolog |
| 1 | *oqxB* | resistance-nodulation-cell division (RND) antibiotic efflux pump | 100 | 100 | Perfect | homolog |
| 1 | CRP | resistance-nodulation-cell division (RND) antibiotic efflux pump | 99.05 | 100 | Strict | homolog |
| 1 | *E. coli* EF-Tu mutants | elfamycin resistant EF-Tu | 97.97 | 96.33 | Strict | variant |
| 1 | *K. pneumoniae acrA* | resistance-nodulation-cell division (RND) antibiotic efflux pump | 95.24 | 99.75 | Strict | homolog |
| 1 | UhpT | Escherichia coli UhpT with mutation conferring resistance to fosfomycin | 95.03 | 100 | Strict | variant |
| 1 | *patA* | ATP-binding cassette (ABC) antibiotic efflux pump | 94.77 | 100 | Strict | homolog |
| 2 | *vgaC* | ABC-F ATP-binding cassette ribosomal protection protein | 94.59 | 100 | Strict | homolog |
| 1 | *E. coli parC* | fluoroquinolone self-resistant *parC*; fluoroquinolone resistant *parC* | 94.41 | 100 | Strict | variant |
| 1 | *K.pneumoniae* OmpK37 | General Bacterial Porin with reduced permeability to beta-lactams | 94.27 | 102.67 | Loose | homolog |
| 1 | *emrB* | major facilitator superfamily (MFS) antibiotic efflux pump | 94.02 | 100 | Strict | homolog |
| 1 | *msbA** | ATP-binding cassette (ABC) antibiotic efflux pump | 92.78 | 100 | Strict | homolog |
| 1 | *marA** | resistance-nodulation-cell division (RND) antibiotic efflux pump; General Bacterial Porin with reduced permeability to beta-lactams | 92.74 | 97.64 | Strict | homolog |
| 1 | *emrR* | major facilitator superfamily (MFS) antibiotic efflux pump | 92.57 | 100 | Strict | homolog |
| 1 | *E. coli gyrA* | triclosan resistant *gyrA* | 92.11 | 100.23 | Strict | variant |
| 1 | *baeR* | resistance-nodulation-cell division (RND) antibiotic efflux pump | 91.67 | 100 | Strict | homolog |
| 1 | *mdtC* | resistance-nodulation-cell division (RND) antibiotic efflux pump | 91.61 | 100 | Strict | homolog |
| 1 | *acrB* | resistance-nodulation-cell division (RND) antibiotic efflux pump | 91.42 | 99.9 | Strict | homolog |
| 1 | *mdtB* | resistance-nodulation-cell division (RND) antibiotic efflux pump | 90 | 100 | Strict | homolog |
| 1 | *aadA* | ANT(3'') | 87.11 | 98.48 | Strict | homolog |
| 1 | *E.coli mdfA* | major facilitator superfamily (MFS) antibiotic efflux pump | 85.61 | 100 | Strict | homolog |
| 1 | AAC(6')-IIb | AAC(6') | 84.62 | 250 | Loose | homolog |
| 1 | PmrF* | pmr phosphoethanolamine transferase | 83.69 | 101.55 | Strict | homolog |
| 1 | CTX-M-45 | CTX-M beta-lactamase | 83.33 | 93.08 | Loose | homolog |
| 1 | *E. coli marR* | resistance-nodulation-cell division (RND) antibiotic efflux pump | 83.33 | 100 | Strict | overexpression |
| 1 | *cphA8** | CphA beta-lactamase | 81.82 | 215.75 | Loose | homolog |
| 1 | H-NS | major facilitator superfamily (MFS) antibiotic efflux pump; resistance-nodulation-cell division (RND) antibiotic efflux pump | 81.82 | 78.1 | Loose | homolog |
| 2 | *arr-5** | rifampin ADP-ribosyltransferase (Arr) | 81.82 | 70.67 | Loose | homolog |
| 1 | *tet(35)** | ATP-binding cassette (ABC) antibiotic efflux pump | 81.82 | 72.09 | Loose | homolog |
| 1 | YojI | ATP-binding cassette (ABC) antibiotic efflux pump | 78.57 | 55.03 | Loose | homolog |
| 1 | SHV-41 | SHV beta-lactamase | 76.92 | 103.15 | Loose | homolog |
| 1 | FosA2 | fosfomycin thiol transferase | 71.32 | 98.58 | Strict | homolog |

*Seq: the number represents chromosome or plasmid genome as shown in Table S2.

*: These genes could not be identified readily from Nanopore outputs and were found in hybrid assembly genome only.

Supplementary Table S5. Seventy-seven virulence factors identified based on hybrid assembly of sample 4.

| Gene | Class | Function | Identity (%) | Qcov^*^ (%) |
| --- | --- | --- | --- | --- |
| *mrkH* | Type 3 fimbriae | transcriptional activator | 100 | 99 |
| *mrkF* | Type 3 fimbriae | type 3 fimbrial minor pilin subunit MrkF | 100 | 99 |
| *mrkC* | Type 3 fimbriae | fimbrial biogenesis outer membrane usher protein *mrkC* precursor | 100 | 99 |
| *mrkB* | Type 3 fimbriae | fimbrial chaperone protein *mrkB* precursor | 100 | 99 |
| *fimE* | Type I fimbriae | tyrosine recombinase | 100 | 99 |
| *fimA* | Type I fimbriae | type 1 major fimbrial subunit precursor | 100 | 99 |
| *fepC* | Ent | iron-enterobactin transporter ATP-binding protein | 100 | 99 |
| *fepG* | Ent | iron-enterobactin transporter permease | 100 | 99 |
| *ybdA* | Ent | enterobactin exporter EntS | 100 | 99 |
| *vipA/tssB* | T6SS | type VI secretion system contractile sheath small subunit VipA | 100 | 99 |
| *vipB/tssC* | T6SS | type VI secretion system contractile sheath large subunit VipB | 100 | 99 |
| *vasE/tssK* | T6SS | type VI secretion system baseplate subunit TssK | 100 | 99 |
| *dotU/tssL* | T6SS | type VI secretion system protein, DotU/TssL family | 100 | 99 |
| *hcp/tssD* | T6SS | type VI secretion system protein, Hcp family | 100 | 99 |
| *clpV/tssH* | T6SS | type VI secretion system ATPase TssH | 100 | 99 |
| *tli1* | T6SS | type VI secretion system immunity protein | 100 | 99 |
| *tle1* | T6SS | type VI secretion system effector | 100 | 99 |
| KPHS_23120 | T6SS | PaaR repeat-containing protein | 100 | 99 |
| *icmF/tssM* | T6SS | type VI secretion protein TssM | 100 | 99 |
| *impA/tssA* | T6SS | type VI secretion system protein TssA | 100 | 99 |
| *tssF* | T6SS | type VI secretion system baseplate subunit TssF | 100 | 99 |
| *tssG* | T6SS | type VI secretion system baseplate subunit TssG | 100 | 99 |
| *sciN/tssJ* | T6SS | type VI secretion system lipoprotein TssJ | 100 | 99 |
| *ybtS* | Yersiniabactin | salicylate synthase Irp9 | 100 | 99 |
| *ybtX* | Yersiniabactin | putative signal transducer | 100 | 99 |
| *ybtP* | Ybt | yersiniabactin ABC transporter ATP-binding/permease protein YbtP | 100 | 99 |
| *ybtA* | Ybt | yersiniabactin transcriptional regulator YbtA | 100 | 99 |
| *ybtU* | Yersiniabactin | yersiniabactin biosynthetic protein YbtU | 100 | 99 |
| *ybtE* | Yersiniabactin | yersiniabactin siderophore biosynthetic protein | 100 | 99 |
| *galF* | Capsule | UTP-glucose-1-phosphate uridylyltransferase subunit GalF | 100 | 99 |
| *rcsB* | RcsAB | transcriptional regulator RcsB | 100 | 99 |
| *acrB* | AcrAB | acriflavine resistance protein B | 99.9 | 99 |
| *vgrG/tssI* | T6SS | type VI secretion system tip protein VgrG | 99.87 | 99 |
| *fyuA* | Ybt | yersiniabactin receptor FyuA | 99.85 | 99 |
| *ybtQ* | Yersiniabactin | inner membrane ABC-transporter YbtQ | 99.83 | 99 |
| *irp1* | Yersiniabactin | yersiniabactin biosynthetic protein Irp1 | 99.81 | 99 |
| *gnd* | Capsule | 6-phosphogluconate dehydrogenase | 99.79 | 99 |
| *acrA* | AcrAB | acriflavine resistance protein A | 99.75 | 99 |
| *ugd* | Capsule | UDP-glucose 6-dehydrogenase | 99.74 | 99 |
| *mrkD* | Type 3 fimbriae | fimbrial adhesin protein precursor MrkD | 99.7 | 99 |
| *fepD* | Ent | iron-enterobactin transporter membrane protein | 99.7 | 99 |
| *fimH* | Type I fimbriae | type 1 fimbrial adhesin precursor | 99.67 | 99 |
| *fimD* | Type I fimbriae | outer membrane usher protein | 99.66 | 99 |
| *entE* | Ent | enterobactin synthase subunit E | 99.63 | 99 |
| *entF* | Ent | enterobactin synthase subunit F | 99.61 | 99 |
| *mrkJ* | Type 3 fimbriae | phosphodiesterase | 99.58 | 99 |
| *irp2* | Yersiniabactin | yersiniabactin biosynthetic protein Irp2 | 99.56 | 99 |
| *rcsA* | RcsAB | transcriptional activator for ctr capsule biosynthesis | 99.52 | 99 |
| *mrkA* | Type 3 fimbriae | type 3 fimbrial major pilin subunit MrkA | 99.5 | 99 |
| *fimB* | Type I fimbriae | tyrosine recombinase | 99.5 | 99 |
| *mrkI* | Type 3 fimbriae | LuxR family regulatory protein | 99.47 | 99 |
| *fimG* | Type I fimbriae | type 1 fimbrial minor component | 99.4 | 99 |
| *fepB* | Ent | iron-enterobactin transporter periplasmic binding protein | 99.37 | 99 |
| *iroE* | Sal | siderophore esterase IroE | 99.35 | 99 |
| *fepA* | Ent | outer membrane receptor FepA | 99.33 | 99 |
| *entB* | Ent | 2,3-dihydro-2,3-dihydroxybenzoate synthetase, isochroismatase | 99.29 | 99 |
| *ybtT* | Ybt | yersiniabactin biosynthesis thioesterase YbtT | 99.25 | 99 |
| *entA* | Ent | 2,3-dihydroxybenzoate-2,3-dehydrogenase | 99.2 | 99 |
| *fimC* | Type I fimbriae | periplasmic chaperone | 99.15 | 99 |
| *fes* | Ent | enterobactin/ferric enterobactin esterase | 99 | 99 |
| *fimK* | Type I fimbriae | transcriptional regulator | 98.96 | 87 |
| *entC* | Ent | isochorismate synthase | 98.72 | 99 |
| *fimI* | Type I fimbriae | type 1 pilus biosynthesis fimbrial protein | 98.31 | 99 |
| *fimF* | Type I fimbriae | type 1 fimbrial minor component | 98.29 | 99 |
| *wzi* | Capsule | surface assembly of capsule | 96.86 | 99 |
| *yagZ/ecpA* | ECP | *E. coli* common pilus structural subunit EcpA | 95.9 | 99 |
| *cpsACP* | Capsule | phosphatase PAP2 family protein | 95.69 | 99 |
| *yagW/ecpD* | ECP | polymerized tip adhesin of ECP fibers | 94.33 | 99 |
| *wza** | Capsule | capsule polysaccharide export protein precursor | 93.63 | 99 |
| *yagX/ecpC* | ECP | *E. coli* common pilus usher EcpC | 93.58 | 99 |
| *ykgK/ecpR* | ECP | regulator protein EcpR | 90.56 | 99 |
| *yagY/ecpB* | ECP | *E. coli* common pilus chaperone EcpB | 90.09 | 99 |
| *yagV/ecpE* | ECP | *E. coli* common pilus chaperone EcpE | 86.86 | 99 |
| *ompA* | OmpA | outer membrane protein A | 85.11 | 99 |
| *entS** | enterobactin | enterobactin exporter, iron-regulated | 84.54 | 99 |
| *entD* | Ent | enterochelin synthetase component D | 83.98 | 99 |
| *kdsA** | LOS | 2-dehydro-3-deoxyphosphooctonate aldolase | 83.04 | 99 |

*: These genes could not be identified readily from Nanopore outputs and were found in hybrid assembly genome only.

*Qcov: query coverage.

Supplementary Table S6. Top 10 species identified in samples 1-3 based on Kraken2 standard database.

|  | Scientific name | Taxonomy ID | Percent (%)^*^ | No. of clade reads^*^ | No. of reads^*^ |
| --- | --- | --- | --- | --- | --- |
| Sample 1 | *Homo sapiens* | 626765 | 98.49 | 626765 | 626765 |
|  | *Klebsiella pneumoniae* | 245 | 0.05 | 318 | 245 |
|  | *Clostridioides difficile* | 7 | 0 | 8 | 7 |
|  | *Escherichia coli* | 4 | 0 | 8 | 4 |
|  | *Neurospora crassa* | 0 | 0 | 7 | 0 |
|  | *Botrytis cinerea* | 0 | 0 | 7 | 0 |
|  | *Staphylococcus aureus* | 5 | 0 | 5 | 5 |
|  | *Plasmodium berghei* | 0 | 0 | 5 | 0 |
|  | *Nostoc sp. NIES-4103* | 4 | 0 | 4 | 4 |
|  | *Bacillus thuringiensis* | 2 | 0 | 4 | 2 |
| Sample 2 | *Klebsiella pneumoniae* | 573 | 65.66 | 393324 | 287838 |
|  | *Homo sapiens* | 9606 | 7.34 | 43999 | 43999 |
|  | *Klebsiella aerogenes* | 548 | 2.36 | 14157 | 13864 |
|  | *Escherichia coli* | 562 | 1.52 | 9089 | 6210 |
|  | *Klebsiella variicola* | 244366 | 1.06 | 6380 | 6133 |
|  | *Klebsiella quasipneumoniae* | 1463165 | 0.57 | 3402 | 2762 |
|  | *Salmonella enterica* | 28901 | 0.4 | 2368 | 694 |
|  | *Klebsiella oxytoca* | 571 | 0.31 | 1877 | 1865 |
|  | *Klebsiella sp. WCHKl090001* | 2153354 | 0.18 | 1076 | 1076 |
|  | *Klebsiella michiganensis* | 1134687 | 0.15 | 885 | 755 |
| Sample 3 | *Klebsiella pneumoniae* | 573 | 62.01 | 284080 | 220315 |
|  | *Klebsiella aerogenes* | 548 | 2.54 | 11652 | 11450 |
|  | *Homo sapiens* | 9606 | 2.09 | 9565 | 9565 |
|  | *Escherichia coli* | 562 | 1.87 | 8563 | 6283 |
|  | *Klebsiella variicola* | 244366 | 1.18 | 5386 | 5165 |
|  | *Klebsiella quasipneumoniae* | 1463165 | 0.67 | 3064 | 2529 |
|  | *Salmonella enterica* | 28901 | 0.53 | 2439 | 725 |
|  | *Klebsiella oxytoca* | 571 | 0.33 | 1499 | 1484 |
|  | *Klebsiella sp. WCHKl090001* | 2153354 | 0.21 | 943 | 943 |
|  | *Klebsiella michiganensis* | 1134687 | 0.17 | 780 | 679 |
| *Percent of reads: Percentage of reads covered by the clade rooted at this taxon. | | | | | |
| *No. of clade reads: Number of reads covered by the clade rooted at this taxon. | | | | | |
| *No. of reads: Number of reads assigned directly to this taxon. | | | | | |

Supplementary Table S7: Two reliable *K. pneumoniae* related reads detected after sequencing started for 3.5min and 10.4min in sample 1

| Read ID | Start time | Duration | Passes filtering | Read length (bp) |
| --- | --- | --- | --- | --- |
| 374d0d1f-a1d6-42da-8f1b-12f988d05a87 | 212.77375 | 12.25 | TRUE | 4413 |
| 4dc720bd-387f-4f35-a81c-c120e563f1b3 | 626.62725 | 14.068 | TRUE | 4581 |

Supplementary Table S8: Consistency of resistance genes and virulence factors identified between direct Nanopore fastq mapping and hybrid assembly.

| Type | Sample | No. of genes | <= 20h | | <=2h | |
| --- | --- | --- | --- | --- | --- | --- |
|  |  |  | Gene family* | Gene* | Gene family | Gene |
| AMR genes | Sample 1 | 8 | 7/34 (20.6%) | 4/34 (11.8%) | 2/34 (5.9%) | 2/34 (5.9%) |
|  | Sample 2 | 70 | 28/34 (82.4%) | 21/34 (61.8%) | 28/34 (82.4%) | 21/34 (61.8%) |
|  | Sample 3 | 62 | 28/34 (82.4%) | 21/34 (61.8%) | 28/34 (82.4%) | 21/34 (61.8%) |
|  | Sample 4 | 60 | 28/34 (82.4%) | 22/34 (64.7%) | 28/34 (82.4%) | 22/34 (64.7%) |
| Virulence factors* | Sample 1 | 12 | 11/77 (14.3%) | | 11/77 (14.3%) | |
|  | Sample 2 | 77 | 74/77 (96.1%) | | 74/77 (96.1%) | |
|  | Sample 3 | 75 | 74/77 (96.1%) | | 74/77 (96.1%) | |
|  | Sample 4 | 77 | 74/77 (96.1%) | | 74/77 (96.1%) | |

*Gene family: The numerator is the total number of antibiotic resistance genes or virulence factors belonging to the same gene family identified based on diret Nanopore fastq mapping-based methods, while the denominator is these identified through hybrid assembly-based methods; the percentage is in brackets.

*Gene: The numerator is the total number of antibiotic resistance genes or virulence factors identified based on diret Nanopore fastq mapping-based methods, while the denominator is these identified through hybrid assembly-based methods; the percentage is in brackets.

*Virulence factors: Gene family data are not shown.

No. number; AMR: antimicrobial resistance genes.
